# Detection and Functional Evaluation of the P2X7 Receptor in hiPSC Derived Neurons and Microglia-Like Cells

**DOI:** 10.3389/fnmol.2021.793769

**Published:** 2022-01-12

**Authors:** Linda Francistiová, Kinga Vörös, Zsófia Lovász, András Dinnyés, Julianna Kobolák

**Affiliations:** ^1^Biotalentum Ltd., Gödöllő, Hungary; ^2^Department of Physiology and Animal Health, Institute of Physiology and Animal Health, Hungarian University of Agriculture and Life Sciences, Gödöllő, Hungary; ^3^HCEMM-USZ Stem Cell Research Group, Hungarian Centre of Excellence for Molecular Medicine, Szeged, Hungary; ^4^Department of Cell Biology and Molecular Medicine, University of Szeged, Szeged, Hungary; ^5^College of Life Sciences, Sichuan University, Chengdu, China

**Keywords:** P2X7 receptor, induced pluripotent stem cell, neuron, microglia, Alzheimer’s disease

## Abstract

A large body of evidence suggests the involvement of the ATP-gated purinergic receptor P2X7 (P2X7R) in neurodegenerative diseases, including Alzheimer’s disease. While it is well-described to be present and functional on microglia cells contributing to inflammatory responses, some reports suggest a neuronal expression of the receptor as well. Here, we present experimental results showing P2X7 receptors to be expressed on human hiPSC-derived microglia-like cells, hiPSC-derived neuronal progenitors and hiPSC-derived matured neuronal cells. By applying cell surface protein detection assays, we show that P2X7R is not localized on the cell membrane, despite being detected in neuronal cells and thus may not be available for directly mediating neurotoxicity. On hiPSC-derived microglia-like cells, a clear membranous expression was detected. Additionally, we have not observed differences in P2X7R functions between control and familial Alzheimer’s disease patient-derived neuronal cells. Functional assays employing a P2X7R antagonist JNJ 47965567 confirm these findings by showing P2X7R-dependent modulation of microglia-like cells viability upon treatment with P2X7R agonists ATP and BzATP, while the same effect was absent from neuronal cells. Since the majority of P2X7R research was done on rodent models, our work on human hiPSC-derived cells presents a valuable contribution to the field, extending the work on animal models to the human cellular system and toward clinical translation.

## Introduction

Neuroinflammation is one of the major underlying pathologies of many neuropathological conditions, including Alzheimer’s disease (AD). It results from various causes such as tissue damage, pathogen infiltration, autoimmune conditions, deposition of pathologic proteins such as amyloid-β, stress, and neuronal hyperexcitability during seizures (Yang and Zhou, 2019). Subsequently, neuroinflammation leads to the biosynthesis and release of various signaling molecules, including cytokines, chemokines, and reactive oxygen species (ROS) (Heneka et al., 2015). Neuroinflammation is an essential protective process in the central nervous system (CNS), as it contributes to the elimination of the negative factor that primarily caused it and the restoration of the normal tissue homeostasis (Gray et al., 2020). However, in the case of prolonged and extended inflammation, like the one that takes place during AD, it can lead to further cellular damage amplifying the existing inflammation and causing irreversible tissue damage (Villegas-Llerena et al., 2016). During neurodegeneration, when neuronal cells are damaged or die, ATP is released into the extracellular environment. This ATP serves as a damage-associated molecular pattern (DAMP) which is detected by microglial cells *via* their purinergic receptors. From amongst both ionotropic (P2X) and metabotropic (P2Y) ATP-gated purinergic receptors (Huang et al., 2019), the P2X7R has attracted most attention due to its unique characteristics such as its low affinity to ATP (Surprenant et al., 1996) and the suggested involvement in a broad range of neurodegenerative diseases such as AD (Martin et al., 2019; Francistiová et al., 2020), epilepsy (Engel et al., 2012; Conte et al., 2020; Morgan et al., 2020), schizophrenia (Calovi et al., 2020), Huntington’s disease (Ollà et al., 2020) and many others. Moreover, another property of the P2X7R is its ability to induce macropore formation upon exposure to high concentrations of ATP (Di Virgilio et al., 2018). The formation of such a macropore in the cell membrane induces cell death and can be experimentally demonstrated by the uptake of large molecules, e.g., Yo-Pro-1 by the cells (Virginio et al., 1999; Faria et al., 2005).

Since P2X7R is mainly expressed on immune cells, it is involved mostly in the context of immune system-related effects. In the CNS parenchyma, the immune cells are represented by microglia—the resident immune cell of the CNS. These cells are involved in many physiological processes, such as developmental synaptic pruning (Paolicelli et al., 2011; Schafer et al., 2012; Parkhurst et al., 2013) and immune surveillance during adulthood (Sierra et al., 2014; Ransohoff and Khoury, 2016). The expression and activity of the P2X7R on microglia have been widely examined and validated (Monif et al., 2009; Amadio et al., 2017; Janks et al., 2018). The P2X7R was also suggested in the pathogenesis of Alzheimer’s disease, as a general driver of neuroinflammation (McLarnon et al., 2006; Sáez-Orellana et al., 2016), amyloid peptide-dependent neuroinflammation (Martínez-Frailes et al., 2019) or *via* modulation of chemokine signaling (Martin et al., 2018).

While the expression of the P2X7R on neurons has also been suggested (Deuchars et al., 2001; Illes et al., 2017; Miras-Portugal et al., 2017; Dragić et al., 2021; Ortega et al., 2021), as well as P2RX7 mRNA presence was detected in excitatory neuronal cells (Metzger et al., 2017; Hodge et al., 2019), an irrefutable conclusion on P2X7R protein distribution is not yet available. One of the reasons behind the difficulties in detecting the P2X7R on neurons is the limitations in the detection methods, such as the lack of sensitive and specific anti- P2X7R antibodies (Nicke et al., 2009; Kaczmarek-Hajek et al., 2018). Another way to examine the presence of the receptor on cells is by performing pharmacological studies. One of the most reliable and specific P2X7R antagonists with high affinity is JNJ 47965567, which has been used successfully in assessing the functionality of the rodent P2X7R. The high selectivity of this antagonist was probed against a panel of 50 other receptors and thus can be considered reliable for *in vitro* pharmacological assays (Bhattacharya et al., 2013).

Human induced pluripotent stem cells (hiPSCs) are generated from somatic cells *via* genetic reprogramming. They resemble embryonic stem cells and can be differentiated into all three germ layers (endoderm, ectoderm, and mesoderm) and can give rise to any type of human somatic cells under proper signals and culture conditions (Takahashi et al., 2007; Shi et al., 2017). Over the past decade, hiPSC technology has become broadly used and presents an invaluable tool in connecting the biological data obtained from rodent models to human diseases. hiPSCs are particularly useful in modeling otherwise hardly accessible tissues, like the human brain (Chambers et al., 2009; Nicholas et al., 2013). Protocols for the generation of neurons and astroglia from hiPSC are readily available and utilized routinely in several laboratories worldwide, including ours (Zhou et al., 2016; Chandrasekaran et al., 2017; Ochalek et al., 2017; Lo Giudice et al., 2019). However, microglia generation from hiPSCs has been problematic for a long time due to the microglia’s unique yolk sac origin. Currently, there are several well-established protocols available for the generation of microglia-like cells (Muffat et al., 2016; Abud et al., 2017; Douvaras et al., 2017; Haenseler et al., 2017; Pandya et al., 2017; Brownjohn et al., 2018; McQuade et al., 2018).

In the present study, we investigated the expression and function of the P2X7R in hiPSC-derived cortical type neuronal cells obtained from a healthy donor and from a patient with familial Alzheimer’s disease, as well as in healthy donor-obtained hiPSC-derived microglia-like cells. Using immunocytochemistry and immunoblot detection, we demonstrate the expression and localization of P2X7R in neuronal and microglia-like cells. Furthermore, by employing pharmacological assays using the highly specific P2X7R antagonist JNJ 47965567, we investigated the activation of P2X7R by administering ATP and BzATP on both cell types. Thus, the results of our study show for the first time P2X7R expression and function on different hiPSC-derived cell types of the CNS.

## Materials and Methods

### Human iPSC Culture

In the present study, hiPSCs, derived from a healthy Caucasian female (35 years old) (here named as Ctrl) (Ochalek et al., 2017), and the PSEN1 (p.Val89Leu) mutant familial Alzheimer’s disease female patient (55 years old) (here named as fAD) (Nemes et al., 2016), which we characterized in our previous studies (Ochalek et al., 2017; Lo Giudice et al., 2019) were used for neuronal and microglia (control cell line) differentiation. Human hiPSCs were cultured on BD Matrigel™ matrix (BD Biosciences, Franklin Lakes, NJ, United States) with mTeSR™1 medium (Stem Cell Technologies, Vancouver, Canada), using Gentle Cell Dissociation Reagent for passages, according to the manufacturer’s instruction. Cells were cultured at 37°C in a humidified atmosphere containing 5% CO_2_. For mycoplasma screening, the Venor^®^GeM-Advance (Minerva Biolabs) Mycoplasma Detection Kit was used according to the manufacturer’s protocol in every fifth passage during maintenance and before freezing.

### hiPSC-Derived Neuronal Cell Differentiation and Cultivation

Neural progenitor cells (NPCs) were generated from each of the human hiPSCs by dual inhibition of SMAD signaling pathway using LDN193189 and SB431542 (Chambers et al., 2009) as we described and characterized the neuronal differentiation recently (Ochalek et al., 2017).

To generate human neurons, frozen stock of NPCs were thawed and plated on the POL/L coated (0.002%/2 μg/cm^2^) dishes and cultured in Neural Maintenance Medium (NMM) (1:1 (v/v) mixture of Dulbecco’s Modified Eagle’s/F12 and Neurobasal-A medium, 1x N-2 Supplement, 1x B-27 Supplement, 1x non-essential amino acids (NEAA), 2 mM L-Glutamine, 50 U/ml Penicillin/Streptomycin), supplemented with 0.2 mM ascorbic acid (AA) and 25 μM 2-Mercaptoethanol at a seeding density of 40,000 cells/cm^2^ for immunocytochemistry (ICC) and 100,000 cells/cm^2^ for Western blot (WB) experiments. The medium was changed every 3–4 days during the terminal differentiation, up to 9 weeks.

### Microglia-Like Cell Generation and Cultivation

For microglia-like cells generation, the protocol of van Wilgenburg et al. (2013) was followed. Briefly, hiPSCs obtained from healthy donor (same cell line was used for control neurons generation) were plated on 96-well low attachment plates and grown in mTeSR1 (STEMCELL Technologies) supplemented with 50 ng/ml BMP-4 (Thermo Fisher Scientific), 50 ng/ml VEGF-165 (Merck), 20 ng/ml SCF (R&D) and RevitaCell Supplement (Thermo Fisher Scientific) to promote myeloid lineage until the formation of 3D embryoid bodies (EBs). After the EBs had formed, they were plated on a tissue-culture 6-well plate in X-VIVO-15 medium (Lonza) supplemented with 1x GlutaMax, 100 U/ml penicillin/streptomycin, 50 μM 2-Mercaptoethanol, 100 ng/ml M-CSF, 25 ng/ml IL-3 (all supplements are from Thermo Fisher Scientific) to induce the production of microglial progenitor cells by the EBs. The 50% of the medium was changed once a week until week 4. After week 4, the EB cultures started to produce microglia precursor cells, which were harvested at weekly intervals and used for further experiments. For monoculture preparation, the harvested microglia precursor cells were seeded on a cell culture plate of desired dimension without any surface pre-coating and cultivated the cells in X-VIVO medium with 100 ng/ml M-CSF.

### Microglia-Neuron Co-cultures

The hiPSC-derived neuronal cultures, differentiated for 42 days (TD42), were grown on coverslips at a 40,000 cells/cm^2^ density in MMM media. Microglial progenitor cells were added to the neuronal cultures at 10,000 cells/cm^2^ density and were kept in co-culture for 14 or 33 days and grown in MMM media. At the end of this cultivation period, cells were fixed with 4% PFA, stained with anti-IBA1 antibody, and the cells’ morphology was analyzed.

### Western Blot

For Western blot analysis, cell pellets were collected from microglia-like cells, NPCs and differentiated neurons at different time points. Cell pellets were lysed using RIPA buffer and supplemented with Halt™ Protease and Phosphatase Inhibitor Cocktail and Pierce™ Universal Nuclease for Cell Lysis (Thermo Fisher Scientific), sonicated and centrifuged to obtain protein extracts. The concentration of proteins was assessed using the Pierce BCA Protein Assay Kit (Thermo Fisher Scientific). Ten micrograms of proteins were separated on a 10% SDS-polyacrylamide precast gel and transferred to Immun-Blot^®^ PVDF Membrane (Bio-Rad Laboratories). Membranes were blocked with 5% milk in TBS and incubated overnight at 4°C with primary antibodies (anti- P2X7R, APR-004, 1:500, Alomone Labs; anti- P2X7R, sc-514962, 1:1,000, Santa Cruz; anti-GAPDH, G9545, 1:5,000, Sigma; anti-NLRP3 (D4D8T), 15101S, 1:1000, Cell Signaling; anti-NESTIN, MAB5326, 1:1,000, Sigma-Aldrich; anti-Integrin α7, sc-27706, 1:1,000, Santa Cruz; anti-HSP27, sc-1049, 1:500, Santa Cruz; anti-CD11c, ab52632, 1:3,000 Abcam), washed (3-times per 10 min) and incubated with secondary antibodies (anti-rabbit-HRP, 7074, 1:2,000, Cell Signaling; anti-mouse-HRP, 7076, 1:2,000, Cell Signaling; anti-goat-HRP, 31400, 1:2,000, Thermo Fisher Scientific) at room temperature for 1 h. The resulting bands were visualized with SuperSignal™ West Dura Extended Duration Substrate by KODAK Gel Logic 1500 Imaging System and Kodak MI SE imaging software. Densitometric analysis of the resulting bands was performed using the Image Studio software (Licor, version 5.2).

### Reverse Transcription Quantitative PCR

Total RNA was isolated from NPCs, differentiated neurons and microglia-like cells at different time points using the RNeasy Plus Mini Kit (Qiagen) according to the manufacturer’s protocol. One microgram of RNA was transcribed using the Maxima First Strand cDNA Synthesis Kit (Thermo Fisher Scientific). The PCR conditions were subjected to 94°C, 3 min, initial denaturation; followed by 40 cycles of 95°C, 5 s, denaturation; 60°C 15 s, annealing and 72°C 30 s, elongation. The amplification reactions were carried out in a total volume of 15 μL using SYBR Green JumpStart Taq ReadyMix (Sigma Aldrich). Reverse transcription quantitative PCR (RT-qPCR) was run on the Rotor-Gene Q 5plex Platform (QIAGEN) using gene-specific oligonucleotide primers. Human GAPDH was used as a reference gene. The data of three replicates were analyzed for each gene using the REST software (2009 V2.0.13).

Gene-specific primers were designed using the Primer3 software (Rozen and Skaletsky, 2000), specified with mFOLD software (Zuker, 2003) and Primer-BLAST software (Ye et al., 2012). Primers were optimized using twofold serial dilution standard curves. For reference, gene GAPDH was used. Primers were validated using a cortical reference RNA (Human Brain, Cerebral Cortex Total RNA (636561, Clontech).

### Immunocytochemistry

Cells were cultured on glass coverslips. At the required time, they were fixed in 4% paraformaldehyde (PFA) for 15 min at room temperature (RT), washed three times with PBS and permeabilized with 0.2% Triton X-100 in PBS. Next, cells were blocked with a blocking buffer (5% bovine serum albumin in 0.1% Tween-PBS) for 1 h at RT. Primary antibodies were applied overnight at 4°C at dilutions 1:500 P2X7R (APR-004, Alomone Labs), 1:500 beta-III-tubulin (802001, BioLegend), 1:500 IBA1 (ab5076, Abcam), 1:1,000 Nestin (MAB5326, Sigma), 1:1,000 CD11c (ab52632, Abcam), GFAP 1:500 (MA5-12023, Thermo Fisher Scientific), 1:500 TMEM119 (PA5-62505, Thermo Fisher Scientific). Then the cells on coverslips were washed 0.1% Tween-PBS. Subsequently, appropriate secondary antibodies were applied to the cells for 1 h (at RT). Stained cells were mounted with ProLong™ Diamond Antifade Mountant with DAPI (Thermo Fisher Scientific) and analyzed under a fluorescence microscope (Axio Imager system with ApoTome; Carl Zeiss MicroImaging GmbH, Göttingen, Germany) controlled by AxioVision 4.8.1 software (Carl Zeiss MicroImaging GmbH).

### Cell Surface Protein Isolation

For the cell surface proteins isolation, Pierce™ Cell Surface Protein Biotinylation and Isolation Kit (Thermo Fisher Scientific) were used. The application and sample acquisition was performed according to the manufacturer’s instructions. Briefly, Sulfo-NHS-SS-Biotin solution was applied on cell cultures for 30 min at 4°C to ensure biotin binding. Afterward, the cells were collected and lysed. The cell lysate was then mixed with NeutrAvidin™ Agarose on a separation column and incubated for 30 min (at RT) with end-over-end mixing on a rotator to ensure the binding of NeutrAvidin™ to Sulfo-NHS-SS-Biotin-labeled proteins. The column was washed, and the flow-through containing the unlabeled (intracellular) proteins was captured to be used on Western blot (labeled as IC). The column was washed thoroughly (using 500 μL of washing buffer applied on the column, centrifuged to pass the buffer and repeated 3-times), and the Sulfo-NHS-SS-Biotin-labeled proteins were eluted from NeutrAvidin™ *via* treatment with a reducing agent (250 mM DTT). The eluted proteins (membrane-bound) were collected and used for Western blot detection (labeled as M).

### Viability Assay

Control hiPSC-derived NPCs were cultured on 96-well plates at 90,000 cells/cm^2^ density and treated with different concentrations of ATP (A7699, Sigma-Aldrich) and BzATP (B6396, Sigma-Aldrich) and incubated at standard cultivation conditions for 24 h. Alternatively, the cells were pre-treated with P2X7R antagonist JNJ 47965567 (5299, Tocris). The working concentration of JNJ 47965567 was consistently 1 μM. The viability of the cultures was assessed using the PrestoBlue™ Cell Viability Reagent (Invitrogen). The fluorescent signal was measured using the Varioskan Flash Multimode Reader (Thermo Fisher Scientific). The cell survival was represented as a percentage of untreated cells (100% viability), while cells killed by exposure to water (positive control) represented 0% viability and analyzed with Prism 9 software (GraphPad, Software, La Jolla, CA, United States).

### LPS Challenge

The cell cultures of microglia-like cells matured for two weeks were incubated in the presence of 1 μg/mL LPS (Invitrogen) for 24 h under standard cultivating conditions. After 24 h, the cells were washed 1x with PBS and lysates were prepared for Western blot analysis. The Western blots were performed and analyzed as described in previous sections.

### Fractal Analysis

The ramification of the microglia-like cells was analyzed using the fractal analysis as previously described (Morrison et al., 2017), which among others, quantifies cell complexity (fractal dimension, D_*B*_), cell size (density) and rotational variance (lacunarity, λ). Fractal analysis is typically applied to single cells. Therefore, we selected eight representative cells from both early and late microglia-neuron co-cultures. Individual microglia-like cells were made binary in the FIJI software (Schindelin et al., 2012), and any additional structures surrounding the cell of interest were manually excluded from the image. Binary images were then converted to outlines using FIJI, and FracLac plugin was applied (FracLac plugin for FIJI). FracLac applies a box plot protocol that quantifies the number of pixel detail with increasing scale. For density estimation, FracLac generates a measure of the convex hull, which circumscribes each cell outline with a polygon and a circle that bounds the convex hull. Density (cell size) is the ratio between the number of pixels encompassed by the cell outline to the area (in pixels) of the convex hull. Lacunarity measures heterogeneity to complement fractal dimensions in describing complexity. It uses box mass instead of box count—as it is well-described in the FIJI reference guide, section for FracLac https://imagej.nih.gov/ij/plugins/fraclac/FLHelp/Introduction.htm.

### Statistical Analysis

For statistical analysis, GraphPad Prism 9 software was used. Data are presented as means ± standard deviation (SD). Student’s *T*-test or Tukey’s multiple comparisons test were used (as stated in the figures’ description) to determine the statistical differences between the samples. Significance was accepted at *p < 0.05, ^**^p < 0.01, ^***^p < 0.001.

## Results

### Neuronal Cells Differentiation From Human Induced Pluripotent Stem Cells and Their Characterization

**FIGURE 1:**
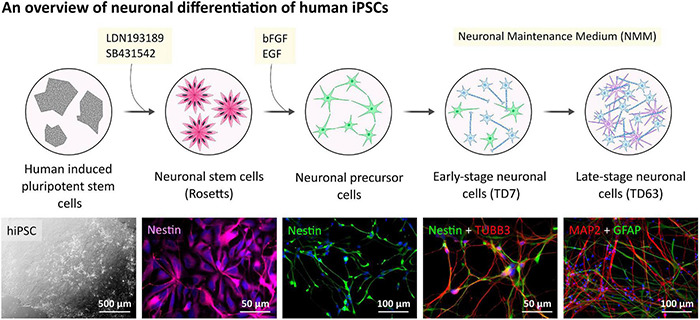
Human hiPSC-derived neuronal cells differentiation and characterization. Outline of the general procedure of hiPSC induction and NPC differentiation toward neuronal cell cultures. Representative phase-contrast image presents hiPSC colonies.

**FIGURE 2:**
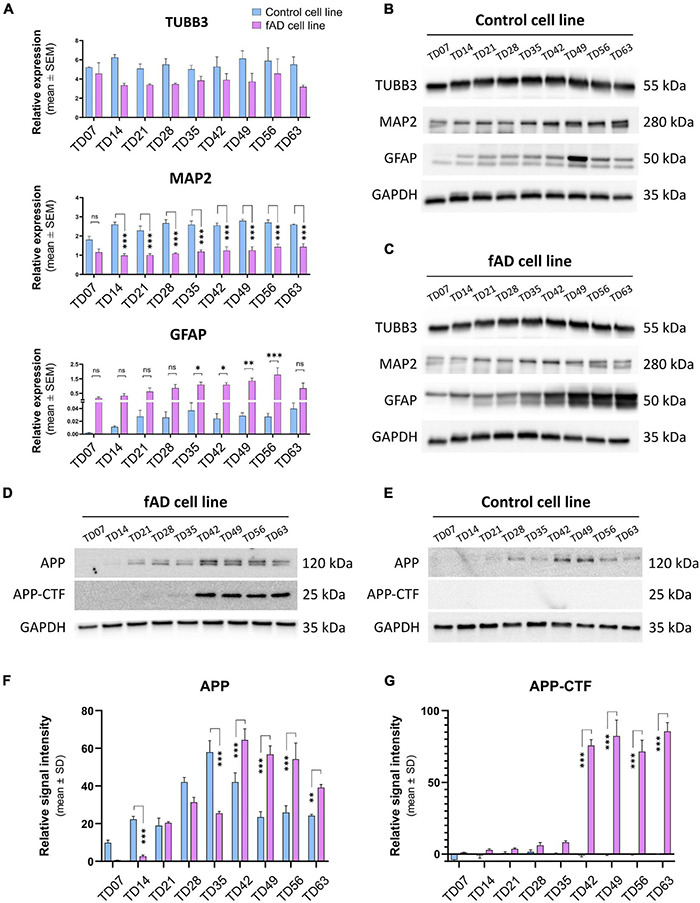
Representative immunostaining of the relevant stages is presenting the basic morphology and expression of main neuronal markers of the cell cultures: Nestin (magenta, or green)—NPC marker; TUBB3 (red) and MAP2 (red) are neuronal markers, and GFAP (green) as an intermediate filament astrocyte marker; nuclei are counterstained with DAPI in all cases (blue). **(A)** RT-qPCR detection of main neuronal (TUBB3 and MAP2) and astrocytic (GFAP) markers on neuronal cultures from TD7 until TD63 stage at weekly intervals. Bars represent the relative expression data (mean ± SEM). Data were normalized with the positive control GAPDH. *p < 0.05; ^**^p < 0.01. The presented analyses are representative of three independent experiments. **(B,C)** Representative Western blot results show the expression of TUBB3, MAP2, and GFAP in the neuronal cultures. 10 μg of protein per lane was used. **(D,E)** Representative Western blots confirm the presence of Alzheimer’s disease-related proteins in our culture system. The presence of the C-terminal fragment of APP (APP-CTF) in the fAD cell cultures but not in the control cell line cultures signifies the ongoing Alzheimer’s disease phenotype. Five micrograms of protein per lane was used. As a positive control, GAPDH was used in all Western blots. **(F,G)** Quantification and comparison of APP and APP-CTF protein expressions in con control and fAD cell lines. Error bars represent the mean ± SD of three measurements. ^***^p < 0.001.

To examine the presence and the functionality of the P2X7R on human neuronal cells, the NPCs were terminally differentiated (Figure 1). The neuronal differentiation of the used hiPSCs, as well as the AD phenotype of the PSEN1 mutant hiPSC line, was characterized and published previously by our laboratory (Ochalek et al., 2017; Lo Giudice et al., 2019). Here, the neuronal cells were kept in culture for 63 days (TD63) to examine the effects of long-term culturing on the AD pathology manifestation and the expression of the P2X7R. While the two cell lines, control and fAD, did not show major differences in the overall differentiation (TUBB3 and MAP2 expression), an increased astroglial differentiation was observed in the fAD cultures, which was demonstrated by RT-qPCR measurements (Figure 2A) and Western blot detection (GFAP expression) (Figures 2B,C). A representative demonstration of the AD pathology is the presence of a 25 kDa C-terminal fragment of the amyloid precursor protein (APP-CTF) in the fAD cell line (Figure 2D) but not in the control cell line (Figure 2E) and the comparison of the two cell lines (Figures 2F,G). Twenty-five kilodaltons sized APP fragment is the typical fragment produced in patients with AD linked to PSEN1 mutations (García-Ayllón et al., 2017).

### Microglia-Like Cell Differentiation From Human Induced Pluripotent Stem Cells and Maturation in Co-culture With Neurons

**FIGURE 3:**
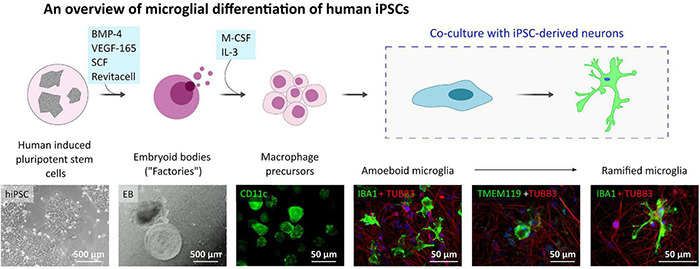
Microglia-like cell culture characterization. Outline of the hiPSC-derived microglia-like cells differentiation and maturation *in vitro*. Microglia-like cells are generated from embryoid bodies (EBs) and further differentiated into macrophage precursors (first two phase contrast images). After adding the early microglia-like cells [CD11c (green) positive cells] to neuronal cultures [immunostained with TUBB3 neuronal marker (red)], they change their shape into amoeboid and later ramified and integrate into the neuronal network [IBA1 positive cells (green)].

**FIGURE 4:**
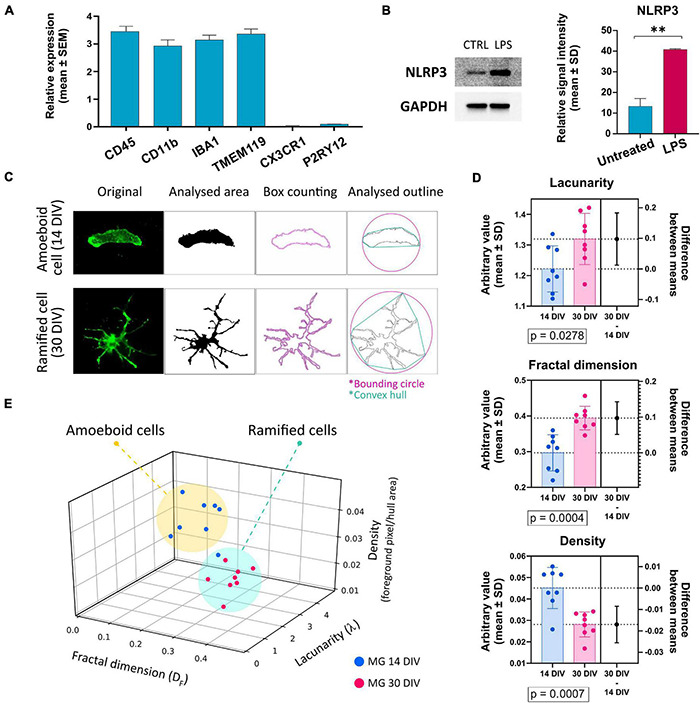
**(A)** Detection of the expression of microglia-specific markers *via* RT-qPCR. Expression values were normalized to GAPDH and calculated as a relative expression value related to the total human cortical mRNA reference sample. Most of the evaluated markers were detected in high amounts, except for CX3CR1 and P2RY12. Error bars represent the mean ± SEM of three measurements performed as technical triplicate. **(B)** Verification of microglia-like cells’ capacity to become activated upon the treatment with 1 μg/ml of LPS. Activation was estimated *via* the detection of NLRP3 protein’s upregulation *via* Western blot. Western blot analysis is representative of three independent experiments. Error bars represent the mean ± SD of three measurements. ^**^p < 0.01. **(C)** Example of the image analysis using FracLac box-counting method for microglia ramification assessment. Original immunocytochemistry pictures were subjected to uniform FIJI protocols, binary picture, and cell outline preparation. Box counting (pink squares along the cell outline), convex hull (turquoise polygon), and bounding circle (pink) were used by FracLac for subsequent calculations. **(D)** Comparison of the individual variables between cells from early and late co-cultures. *n* = 16. **(E)** Plotting the values on a 3D plot based on the relationship between the three variables (fractal dimension, lacunarity, density) distributes the individual data points (representing individual microglia cells) into two populations marked as amoeboid cells and ramified cells. The two groups’ distribution correlates with the time-lengths of microglia-neuron co-cultivations. Error bars represent the mean ± SD. Statistical analysis was performed with an unpaired Student’s *t*-test. *p*-values are indicated below the respective graphs.

Since the P2X7R is well-known to be present and active on phagocytic cells, including microglia cells, in this study, we used microglia-like cells generated from human hiPSC (outlined in Figure 3) following a previously described Embryoid Body (EB)-based 3D differentiation protocol (van Wilgenburg et al., 2013; Haenseler et al., 2017). Detection of microglia-specific markers after 2 weeks showed that the monocultured cells were expressing microglia-specific markers (Figure 4A) such as CD11b, IBA1 and TMEM119. However, relatively high levels of CD45 suggest a similarity to macrophages rather than microglia, which are usually defined as CD45*^low^* or CD45*^int^* (Rangaraju et al., 2018; Honarpisheh et al., 2020). Also, the typical microglial markers CX3CR1 and P2RY12 show low expression levels at this stage. CX3CR1 encodes the receptor for fractalkine, which serves as a main microglia-neuron signaling molecule and is typically expressed in later stages of microgliogenesis (Kierdorf et al., 2013). When the microglia-like cells that had matured for 2 weeks were treated with 1 μg/ml LPS for 24 h they became activated and showed significant upregulation of NLRP3 inflammasome (Figure 4B), which proves the capacity of the cells to become activated and be immunologically engaged.

When microglia precursor cells were co-cultured with neurons, an outstanding integration of the microglia-like cells into the neuronal network and subsequent transformation of the microglia cells’ morphology from round to amoeboid to branched was observed. To compare and quantify the two distinct morphologies, fractal analysis of the cells was conducted (Figures 4C–E), which showed a significant difference in the morphologies of the two populations: amoeboid (early co-cultures—less than 14 days) and ramified (long-term co-cultures—over 30 days) (Figure 4D). Based on the fractal analysis, the analyzed cells were divided into two groups that correlate with the length that microglia cells spent in co-culture with neurons (Figure 4E). These results demonstrate that our hiPSC-derived microglia-like cells well-tolerated co-culturing with hiPSC-derived neurons. Moreover, the ramification and active interweaving of the microglial processes into the network of neuronal cells suggest that hiPSC-derived microglia-like cells acquire a morphology that resembles the resting microglial phenotype.

### P2X7 Receptor Expression in Control and fAD Neuronal Cells and Microglia-Like Cells

**FIGURE 5:**
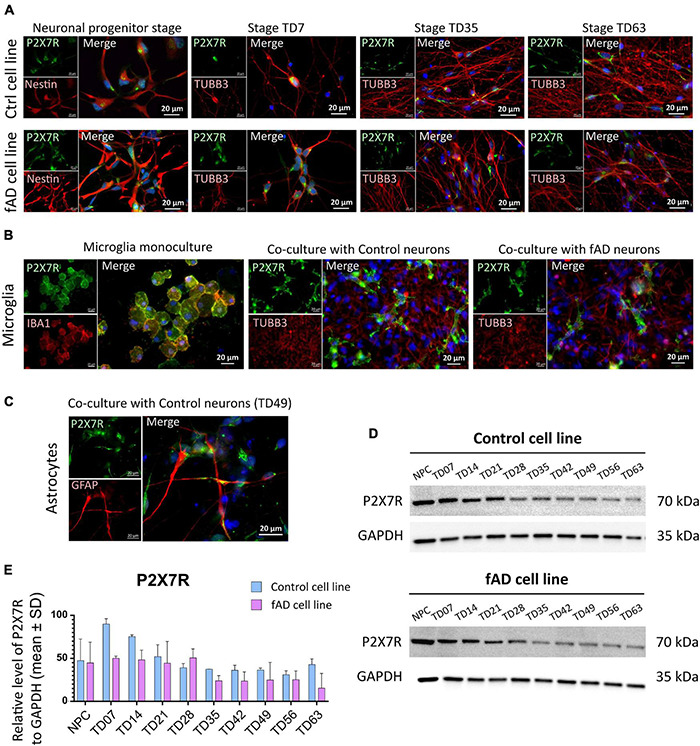
Detection of the P2X7R in hiPSC-derived neuronal and microglia-like cell cultures. Immunocytochemical detection of P2X7R in different differentiation stages of control and fAD hiPSC-derived neurons **(A)** show the presence of the P2X7 receptor (green) in early stages of neuronal differentiation (NPC and TD07) and weaker signal in later differentiation stages (TD35, TD63). In contrast, a strong signal was detected on hiPSC-derived microglia **(B)** in both the early stage of microglial maturation in monoculture, as well as in co-culture with neurons (both control and fAD). Detection of coexpression of P2X7R (green) with GFAP (red) positive astrocytes **(C)**. The pictures are representative of at least three experiments performed on three independent cell cultures. For Western blot analysis 10 μg of protein was loaded on the gel. Representative pictures of the results **(D)** show the presence of a positive signal for P2X7R protein in all neuronal samples, with signal intensity decreasing along with the differentiation time points **(D)**. Densitometric analysis of the Western blot detection illustrates the decrease in signal intensity. The analysis did not show a significant difference between the control and fAD cell line **(E)**. Western blot analysis was performed in biological triplicates and analyzed in at least three technical replicates. Error bars represent the mean ± SD of three measurements.

Immunocytochemical investigation revealed the presence of P2X7R-positive signal in neurons of both control and fAD cell lines in the neuronal progenitor stage, and throughout all the differentiation stages, up to 9 weeks (TD63). However, we have noticed that while in the NPC and early terminal differentiation stage of neurons (TD7), the expression seems to be membranous, in the later differentiation stages, the signal weakened and disappeared from the cell membrane of neurons (positive for TUBB3 in colocalization). Instead, it became strongly centralized in the intracellular compartments of the cells (Figure 5A). In the immunocytochemical experiments, no differences were observed in the P2X7R localization detected on control and fAD cells. The shift from membranous to intracellular staining was similar in both cell lines. In the case of microglia-like cells, a clear P2X7R signal was detected on both monocultured and co-cultivated microglia-like cells. Moreover, the P2X7R’s localization on microglia-like cells was uniformly membranous in all examined conditions (Figure 5B). Additionally, immunocytochemical detection of the P2X7R in GFAP co-stained samples was performed (Figure 5C). Results showed the co-expression of P2X7R and GFAP protein, suggesting the presence of P2X7R on astrocytes as well. When Western blot analysis was performed, a gradual decrease in the signal intensity along the neuronal differentiation time of the P2X7R expression was detected (Figures 5E,D).

### Subcellular Localization of P2X7 Receptor Shows Differences Between the Cell Types

**FIGURE 6:**
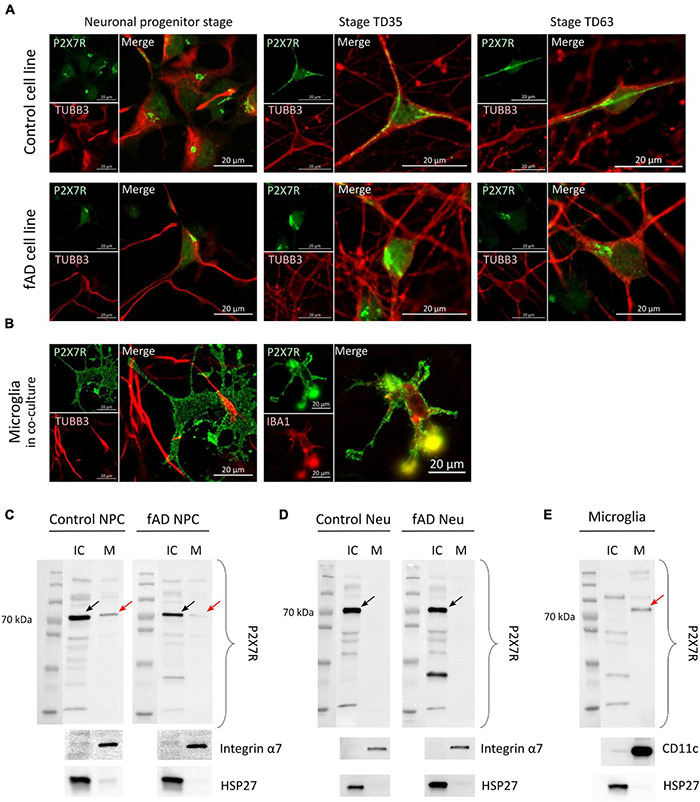
Representative confocal images suggest intracellular localization of the P2X7 receptor’s signal (in green) in Ctrl and fAD neuronal cells (NPC or neurons; labeled with TUBB3 (in red) **(A)** and membranous localization in microglia-like cells **(B)**. Each staining is a representative picture of at least six independent experiments. Microglia-like cells were co-stained with IBA1 (red). Scale bar: 20 μm **(C,D)** and **(E)** show representative Western blot results of biotinylation analysis. Arrows indicate the bands representing the canonical ≈72 kDa sized P2X7R localized in the IC (intra-cellular) fraction in the case of Ctrl and fAD neurons’ (TD53) samples **(D)** and the M (membrane) fraction of microglia samples **(E)** and both fractions in NPC samples **(C)**. Red arrows indicate the presence of the P2X7R band in the M fractions. The Integrin α7 **(C,D)** and CD11c **(E)** are plasma membrane proteins present only in the M factions. HSP27 is a nuclear protein present only in the IC fractions **(C–E)**. Integrin α7 and HSP27 were used as controls of the efficiency of biotinylation and the separation of biotinylated proteins. Western blot measurements were per-formed as biological triplicates and a duplicate in the case of microglia-like cells. Ctrl, control; Neu, neuron; NPC, neuronal progenitor cell; IC, intra-cellular; M, membrane.

Proteins of the P2X receptor family are all membrane-bound ion channels, and thus the localization is crucial for their proper ion channel function. To investigate whether the detected P2X7R signal originates from its expected subcellular localization—the cell membrane, high-resolution confocal microscopy was performed on NPCs, TD35 and TD63 neuronal cells from both Ctrl and fAD cells, as well as on hiPSC-derived microglia-like cells (Figures 6A,B). Our results suggest that the signal from the NPCs and neurons is of intracellular origin, while the signal detected from microglia-like cells clearly outlined the whole surface of the cell, indicating membranous expression of the receptor (Figures 6A,B). The intracellular localization of the P2X7R has been reported by others (Gu et al., 2000; Sarti et al., 2021). Therefore, to further investigate the localization of the P2X7R on our cells, cell surface protein biotinylation and isolation assay was performed. The results confirmed the observations from the immunocytochemistry experiments and showed the presence of P2X7R in the fraction containing the intracellular proteins (IC) in the case of neuronal cells examined at stage TD35 (both Ctrl and fAD cell lines) (Figure 6D). In contrast, the receptor was present in the membrane-bound proteins’ fraction (M) in microglia-like cells (Figure 6E) and in NPCs as well (Figure 6C). Altogether, our findings indicate that the P2X7R is localized on the cell membrane of microglia and NPCs but not on the neuronal cells.

### Pharmacological Assays Show Differences in P2X7R-Dependent Cell Viability in Neuronal and Microglia-Like Cells

**FIGURE 7:**
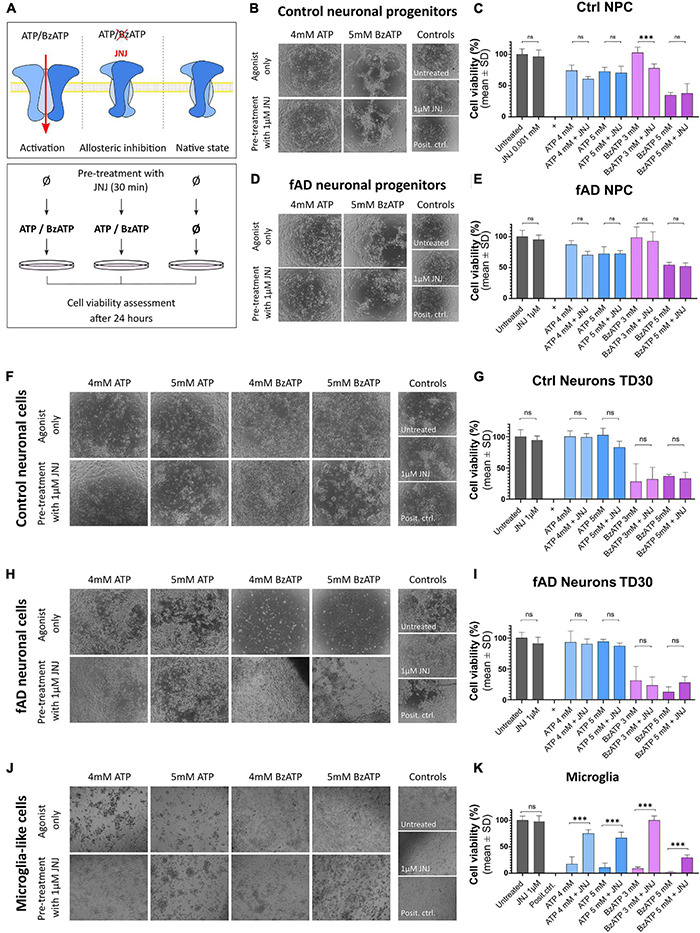
Functional assay results after the application of P2X7R agonists ATP and BzATP alone upon pre-incubation with the highly specific P2X7R antagonist JNJ 47965567. The working concentration of JNJ 47965567 was 1 μM in all conditions. **(A)** Outline of the experimental design. Cells were treated for 24 h with ATP or BzATP in different concentrations to promote P2X7R activation; in the samples treated with the P2X7R antagonist JNJ 47965567, the pre-treatment was performed for 30 min before the application of ATP or BzATP to induce blockage of the receptor. Cell cultures were monitored as representative phase-contrast photographs show. Cell viability was measured using PrestoBlue cell viability assay after the treatment in control **(B,C)** and fAD neuronal pro-genitors **(D,E)**; TD35 differentiation stage control **(F,G)** and fAD neuronal cells **(H,I)** and microglia-like cells differentiated for 2 weeks **(J,K)**. In all graphs, the viability of the untreated cells represents 100% viability, and the viability of positive controls (cells treated with water to induce total cell death) represent 0% viability. All the measured values were normalized to the two controls. The presented data are of two independent experiments with each condition performed in triplicate cultures. Error bars represent the mean ± SD. Statistical analysis was performed by ordinary one-way ANOVA followed by Tukey’s multiple comparisons test; *p*-value ^***^ < 0.001. ns: not significant.

To examine whether the observations from the localization studies are reflected in the functional assays, we performed pharmacological assays (Figure 7A). Control and fAD neuronal cells (at NPC and TD30 stage) and the microglia-like cells (at 14 DIV) were treated with different concentrations of P2X7R agonists ATP and BzATP or pre-treated with the highly specific P2X7R antagonist JNJ 47965567 (Bhattacharya et al., 2013). Our results show that the ATP application had a negative effect on the overall viability of the NPCs (Figures 7B–E) while in the case of the cells treated with a lower concentration of ATP (4 mM) this negative effect was slightly reduced upon the application of the P2X7R antagonist JNJ 47965567. No difference was observed between the control and fAD NPC cultures. The BzATP application had various effects on the NPCs. The viability of the cells treated with lower concentrations of BzATP (3 mM) was not different from the untreated control, but the JNJ 47965567 pre-treated cells showed decreased viability. The viability of the cells treated with higher concentrations of BzATP (5 mM) was significantly decreased, and pre-treatment with JNJ 47965567 had no effect on the measured viability. This effect was similar in both control and fAD cell lines. These effects can be observed on brightfield images, showing very similar cell culture quality in agonist only and pre-treatment with 1 μM JNJ. Neuronal cultures matured for 30 days did not show a decrease in viability upon treatment with ATP (Figures 7F–I), while treatment with BzATP significantly decreased their viability. However, the pre-treatment of the cell cultures with JNJ 47965567 had no effect on the cells’ viability. Viability levels were similar in control and fAD cell lines. In microglia-like cells, different concentrations of both ATP and BzATP treatment of the cells caused a significant decrease in cell viability (Figures 7J,K). In contrast to neuronal cells, pre-treatment of microglia-like cells with JNJ 47965567 significantly increased the cells’ viability. With a close look at the brightfield images, it can be seen that the cell cultures quality changes in accordance with the measured viability. This is demonstrated by better attachment and morphology of the well-surviving cells, while in the cultures with decreased viability, the cells detach from the culture plate and appear as floating debris.

Overall, these results show that in microglia-like cell cultures, ATP and BzATP induced cellular death, which can be effectively prevented by pre-treatment of the cells with P2X7R antagonist JNJ 47965567. This protection against toxicity was not observed in the case of neuronal cells or neuronal progenitor cells.

## Discussion

In the present study, we confirmed that hiPSC-derived neurons and astrocytes provide a well-established and suitable *in vitro* modeling system for studying different CNS-related mechanisms. Moreover, by including hiPSC-derived microglia-like cells in this system and our experiments, we have further improved this CNS *in vitro* model and obtained an important control cell for the P2X7R-related experiments. We presented novel data by investigating the ATP-gated purinergic P2X7R in a human neurodegenerative disease model.

Here, we showed the basic characteristics of the used neuronal cells, including the main AD-pathology-related trait—APP-CTF expression. The AD-phenotype of the neuronal cells used in this study was extensively studied and assessed in our previous published work (Chandrasekaran et al., 2017; Ochalek et al., 2017; Lo Giudice et al., 2019). Previously, we reported the establishment of a hiPSC-based cellular platform able to model *in vitro* the major pathological events of both the familial and the sporadic form of Alzheimer’s disease in neuronal cell cultures. We reported an increased Aβ_42_/Aβ_40_ ratio, TAU hyperphosphorylation and oxidative stress sensitivity (Ochalek et al., 2017), which agreed with others’ earlier results (Penney et al., 2020). Moreover, we reported the modulation of AβPP processing and amyloid secretion upon γ-secretase inhibitor, DAPT and the calcilytic NPS 2143 (acting through CaSR) treatment (Lo Giudice et al., 2019). To increase the similarity of our *in vitro* system to the *in vivo* conditions as much as it is possible, in this study, we included hiPSC-derived microglia-like cells in the experiments. Moreover, the here studied purinergic receptor P2X7, and its functions are best described on immune cells (Wewers and Sarkar, 2009; De Torre-Minguela et al., 2016; Janks et al., 2018). Therefore, the addition of human hiPSC-derived microglia-like cells really serves as an excellent control for our experimental conditions.

In our studies, we showed that our hiPSC-derived microglia-like cells closely resembled the characteristics of *in vivo* microglia cells. The microglia-like cells expressed several microglia-specific markers, including IBA1, CD11b, and TMEM119. The absence of CX3CR1 expression, high levels of CD45, but the presence of other microglial markers might suggest an early developmental stage of the analyzed microglia cells sample. Low expression of the P2RY12 is most probably due to the absence of neuronal cells in the culture, namely the lack of TGF-β signaling (Butovsky et al., 2014; Arnold et al., 2019). For true microglia, however, the expression of CX3CR1 and P2YR12 genes are desired. However, the here presented detection of these markers was performed in cells that were kept in monoculture for easier processing. Thus, microglia did not interact with neuronal cells as it is *in vivo*, which probably resulted in decreased expression of CX3CR1, the fractalkine receptor, which is one of the main mediators of neuron-microglia interaction (Sheridan and Murphy, 2013; Pawelec et al., 2020). It has been proven that the lack of CX3CR1/CX3CL1 signaling leads to loss of the suppression of microglial activation (Bolos et al., 2017), and thus possibly influences the expression of the P2Y12R as well. The P2Y12R is most highly expressed in mature microglia, and its expression levels depend on microglial activation. In “M2” activated microglia, it is upregulated and participates in the chemotaxis of microglial cells toward the source of the injury (Haynes et al., 2006). In contrast, during pro-inflammatory activation of microglia, the expression of P2Y12R is decreased (Moore et al., 2015). Therefore, low levels of CX3CR1 and P2Y12R in our microglia-like cells are likely due to the combination of the early developmental stage of the cells, lack of neuronal signaling and activated/primed state of the cells. Nevertheless, the microglia-like cells respond to activation cues such as LPS treatment which we demonstrated by the detection of NLRP3 levels (Figure 4B). It is well-known that co-culture of microglia with neurons and glia cells improves the identity of the microglia cells to better resemble *in vivo* cells (Grubman et al., 2020). And indeed, our microglia-like cells closely mimic microglial morphology and interactions with neuronal networks when placed in co-culture. In this study, we present the gradual morphological changes from amoeboid to ramified occur upon placing microglia-like cells in co-culture with neuronal cells. These events appears to be similar to those described in re-population studies *in vivo* (Svoboda et al., 2019).

Still, microglia-like cells used in this study had proven themselves appropriate and useful in the detection of P2X7R and related and functional studies. Using immunocytochemistry, cell surface protein isolation assay and functional assays, we demonstrate that in the human hiPSC-derived microglia-like cells, the P2X7R is abundantly present and functional in toxicological studies.

There is an intense discussion regarding the presence and potential function of the P2X7R on neuronal cells (Illes et al., 2017; Miras-Portugal et al., 2017). While P2X7R is a well-described immune receptor, it is questionable whether its expression and, even more importantly, functionality on neuronal cells can be confirmed. Here we showed that the P2X7R could be detected in the human hiPSC-derived neuronal progenitor cells and neuronal cells (both on neurons and astroglia cells). However, only in the case of NPCs we were able to localize the receptor on the membrane and to observe some functionally relevant responses on P2X7R activation. We confirmed the validity of the measurements by using microglia cells. The results confirm the notion of microglia being the primary source of P2X7R in the CNS (Campagno and Mitchell, 2021). Moreover, we detected a gradual decrease of the P2X7R signal during hiPSC-derived neuron’s differentiation in both Western blot detection and ICC visualization. This seems to be in line with the proposed assumption that P2X7R has a role in neurogenesis which is executed by NPCs (Tang and Illes, 2017). Thus the presence of the P2X7R on the NPCs would be in agreement with previously reported research showing the presence and functional importance of P2X7R in NPCs during neurogenesis (Leeson et al., 2018). It would therefore make sense that with the progression of the NPC differentiation toward mature neurons, the P2X7R disappears from the cells’ surface and the overall expression decreases.

Interestingly, a number of articles reported the presence of P2X7R in astrocytes (Kamatsuka et al., 2014; Gao et al., 2017; Khan et al., 2019), including a work on iPSC-derived cells (Kesavan et al., 2021). Some of these reports suggest that the astrocytic P2X7R indirectly regulates neuronal activity (Khan et al., 2019). We were also able to detect the P2X7R signal in astrocytes *via* immunocytochemistry. However, with the tools and approaches currently available to us, it was not possible to separate the neuronal and astrocyte cells and assess the localization and functionality of P2X7R in astrocytes only. A good avenue for future experiments could be the examination of P2X7R in hiPSC-derived pure astrocytes and its interposed impact on neuronal cells. Ideally, a model system consisting of neurons, astrocytes, oligodendrocytes and microglia could possibly provide valuable data about the effects of intercellular communication on P2X7R signaling in the human brain in health and disease.

In the pharmacological assays presented in this study, P2X7R-dependent effects of ATP and BzATP on the microglia-like cells’ viability were observed. These observations are in agreement with the knowledge that the P2X7R is primarily related to immunological events and thus is mainly present on macrophages and microglia (Janks et al., 2018). However, the different protective capacity of the P2X7R antagonist JNJ in the case of 3 and 5 mM BzATP might indicate either that the JNJ concentration was not sufficient to protect the cells from such high BzATP concentrations or that 5 mM BzATP is so far exceeding the physiological range, that the toxic effect is not only the result of P2X7R activation but also other mechanisms (other purinergic receptors’ activation). The observed inability of the P2X7R antagonist JNJ to prevent BzATP-induced cell death of neuronal cells might suggest P2X7R independent activity of BzATP, for example, by activation of other P2X receptors. In the case of neuronal progenitor cells, it is known that the expression of the P2X7R on NPCs can have multiple roles, from phagocytosis in the absence of ATP to modulation of the cells’ proliferation rates, depending on the ATP concentration (Lovelace et al., 2015; Leeson et al., 2018). The fact that some effect was observed in NPCs treated with ATP and BzATP when pre-treatment with JNJ 47965567, is in line with our protein localization study that showed the presence of P2X7R in both intracellular and membrane-bound forms (Figure 6C). We assume that a delicate balance of the ATP/BzATP concentration is needed to consistently regulate these processes in NPCs, while the concentrations used in this study might not satisfy this requirement. Overall, the efficiency of the highly specific P2X7R antagonist JNJ 47965567 implies the involvement of the P2X7R in microglia and in a lesser amount in NPCs, but not in neuronal cells. It is important to note, however, that it could be possible that there is a brain region-dependent variation in P2X7R expression. While the expression dynamics in our cortical-type neuronal cultures suggest a gradual decrease in P2X7R along with the neuronal differentiation, in other brain area-specific neurons, this might differ. It has been shown recently by single-nucleus RNA-sequencing approach, that P2X7R mRNA clusters are present in excitatory, but not in inhibitory neurons (Hodge et al., 2019). Moreover, substantial regional and developmental heterogeneity of protein expression in the brain in health and disease has been reported (Dauth et al., 2017; Collado-Torres et al., 2019; Herrero-Navarro et al., 2021), and therefore P2X7R expression in brain area-specific hiPSC-derived neuronal cultures could be another important direction for future investigation.

In conclusion, in this study, we examined the expression and functionality of the P2X7R in human hiPSC-derived cortical neuronal cultures and microglia-like cells. We demonstrated that P2X7R is expressed in an active form on microglia-like cells and neuronal progenitor cells. While it is possible to detect the receptor in the intracellular compartment of neurons, no P2X7R-specific functional response to excessive amounts of P2X7R agonists (ATP and BzATP) was detected on neurons. Despite being known primarily as a plasma membrane channel, P2X7R has been previously identified in the intracellular compartment. It has been reported, for example, to span the nuclear membrane (Atkinson et al., 2002), participate in phagosomes stabilization by influencing the actin assembly on the surface of the phagosome (Kuehnel et al., 2009) and mitochondria (Sarti et al., 2021). Nevertheless, the intracellular roles of P2X7R are not very well-explored yet.

Thus, we propose that the main cells in the CNS P2X7R signaling pathway are microglia cells. Nevertheless, it would be important to identify the role of the P2X7R in the intracellular compartments of neuronal cells in further studies.

## Data Availability Statement

The original contributions presented in the study are included in the article/supplementary material, further inquiries can be directed to the corresponding author.

## Ethics Statement

The study was reviewed and approved by Hungarian Health Scientific Council (ETT-TUKEB). The generation of the human induced pluripotent stem cell lines and their subsequent use was authorized by the Hungarian Health Scientific Council (ETT-TUKEB) by 17 June 2014, approval letter No. 31203-1/2014/EKU (314/2014). The patients/participants provided their written informed consent to participate in this study.

## Author Contributions

LF prepared the experimental design and performed the experiments, performed cell culturing, Western blots, RT-qPCR, immunocytochemistry, pharmacological and viability assays, analyzed the data, participated in the data interpretation, and drafted the manuscript. ZL and KV generated the microglia-like cells. JK supervised the experimental design, the execution of the experiments, data analysis, and results’ interpretation, participated in the manuscript writing. AD designed the research project, participated in the interpretation of the results, and revised the manuscript. All authors read and approved the final version of the manuscript.

## Conflict of Interest

All authors are (or were at the time of the study) employed by Biotalentum Ltd.

## Publisher’s Note

All claims expressed in this article are solely those of the authors and do not necessarily represent those of their affiliated organizations, or those of the publisher, the editors and the reviewers. Any product that may be evaluated in this article, or claim that may be made by its manufacturer, is not guaranteed or endorsed by the publisher.
